# Correction: Rondinone et al. Extensive Placental Methylation Profiling in Normal Pregnancies. *Int. J. Mol. Sci.* 2021, *22*, 2136

**DOI:** 10.3390/ijms23105298

**Published:** 2022-05-10

**Authors:** Ornella Rondinone, Alessio Murgia, Jole Costanza, Silvia Tabano, Margherita Camanni, Luigi Corsaro, Laura Fontana, Patrizia Colapietro, Luciano Calzari, Silvia Motta, Carlo Santaniello, Tatjana Radaelli, Enrico Ferrazzi, Silvano Bosari, Davide Gentilini, Silvia Maria Sirchia, Monica Miozzo

**Affiliations:** 1 Research Laboratories Coordination Unit, Fondazione IRCCS Ca’ Granda Ospedale Maggiore Policlinico, 20122 Milan, Italy; ornella.rondinone@unimi.it (O.R.); alessio.murgia@policlinico.mi.it (A.M.); jole.costanza@policlinico.mi.it (J.C.); margherita.camanni@policlinico.mi.it (M.C.); laura.fontana@unimi.it (L.F.); silvia.motta2@unimi.it (S.M.); carlo.santaniello@policlinico.mi.it (C.S.); monica.miozzo@unimi.it (M.M.); 2 Medical Genetics, Department of Pathophysiology and Transplantation, Università degli Studi di Milano, 20122 Milan, Italy; silvia.tabano@unimi.it (S.T.); patrizia.colapietro@unimi.it (P.C.); 3 Laboratory of Medical Genetics, Fondazione IRCCS Ca’ Granda Ospedale Maggiore Policlinico, 20122 Milan, Italy; 4 Department of Brain and Behavioral Sciences, Università di Pavia, 27100 Pavia, Italy; luigi.corsaro01@universitadipavia.it; 5 Medical Genetics, Department of Health Sciences, Università degli Studi di Milano, 20122 Milano, Italy; silvia.sirchia@unimi.it; 6 Bioinformatics and Statistical Genomics Unit, Istituto Auxologico Italiano IRCCS, 20095 Cusano Milanino, Italy; luciano.calza@gmail.com; 7 Department of Obstetrics and Gynecology, Fondazione IRCCS Ca’ Granda Ospedale Maggiore Policlinico, 20122 Milan, Italy; tatjana.radaelli@policlinico.mi.it (T.R.); enrico.ferrazzi@policlinico.mi.it (E.F.); 8 Department of Clinical Sciences and Community Health, University of Milan, 20122 Milan, Italy; 9 Scientific Direction, Fondazione IRCCS Ca’ Granda Ospedale Maggiore Policlinico, 20122 Milan, Italy; silvano.bosari@policlinico.mi.it

In the original publication [1], there was a mistake in Table 2 and Table 3 as published. In the *p*-value column, a minus sign was missing at the exponent. The corrected Table 2 and Table 3 appear below. The authors apologize for any inconvenience caused and state that the results and scientific conclusions are unaffected by these changes. The original publication has also been updated.

## Figures and Tables

**Table 2:** Diseases or Functions Annotation from IPA Core Analysis on given gene datasets. The top 10 most significant annotations are shown.

**A**	
**Diseases or Functions Annotation**	***p*-Value**
Leukocyte migration	1.52 × 10^−16^
Cell movement of lymphocytes	5.05 × 10^−14^
Cell movement of mononuclear leukocytes	6.30 × 10^−14^
Quantity of leukocytes	7.50 × 10^−14^
Lymphocyte migration	9.59 × 10^−14^
Quantity of lymphatic system cells	1.97 × 10^−13^
Quantity of lymphocytes	5.29 × 10^−13^
Cell movement of leukocytes	6.80 × 10^−13^
Proliferation of blood cells	1.21 × 10^−12^
Proliferation of immune cells	1.62 × 10^−12^
**B**	
**Diseases or Functions Annotation**	***p*-Value**
Cutaneous melanoma	1.52 × 10^−12^
Skin tumor	5.94 × 10^−12^
Melanoma	1.01 × 10^−11^
Skin cancer	1.20 × 10^−11^
Malignant neuroendocrine neoplasm	2.45 × 10^−7^
Small cell lung carcinoma	3.07 × 10^−7^
Olfactory response	3.73 × 10^−7^
Neuroendocrine tumor	4.48 × 10^−7^
Extrapancreatic neuroendocrine tumor	5.84 × 10^−7^
Blue round small cell tumor	7.16 × 10^−6^

(**A**) Functions identified from gene promoters methylated in the placenta but not in cord blood. (**B**) Functions identified from gene promoters methylated in cord blood but not in the placenta.

**Table 3:** Diseases or Functions Annotations in common between methylation-profiling microarray data and targeted methylation sequencing data (data from IPA Core Analysis).

**A**		
**Diseases or Functions Annotation**	***p*-Value (Microarray)**	***p*-Value (Methyl-Seq)**
Leukocyte migration	2.93 × 10^−26^	4.53 *×* 10^−7^
Quantity of lymphatic system cells	3.30 *×* 10^−25^	4.34 *×* 10^−6^
Quantity of leukocytes	1.51 × 10^−25^	3.42 *×* 10^−6^
Quantity of lymphocytes	1.53 *×* 10^−24^	4.75 *×* 10^−6^
Cell movement of leukocytes	5.69 *×* 10^−23^	1.38 *×* 10^−10^
Activation of cells	2.86 *×* 10^−22^	5.05 *×* 10^−14^
Migration of cells	1.08 *×* 10^−21^	7.35 *×* 10^−7^
Proliferation of lymphatic system cells	1.68 *×* 10^−21^	1.62 *×* 10^−6^
Proliferation of lymphocytes	5.96 *×* 10^−21^	2.09 *×* 10^−6^
Proliferation of immune cells	3.46 *×* 10^−20^	1.58 *×* 10^−6^
**B**		
**Diseases or Functions Annotation**	***p*-Value (Microarray)**	***p*-Value (Methyl-Seq)**
Cross-linkage of protein	1.60 × 10^−4^	8.28 × 10^−4^
Skin carcinoma	7.37 × 10^−4^	3.95 × 10^−3^
Skin squamous cell carcinoma	9.61 × 10^−4^	2.69 × 10^−4^
Relaxation of heart ventricle	1.03 × 10^−3^	5.24 × 10^−3^
Surface area of ventricular myocytes	1.22 × 10^−3^	6.15 × 10^−3^
Gastro-esophageal carcinoma	3.77 × 10^−3^	2.78 × 10^−^^3^
Chemosensitivity of glioblastoma cells	4.03 × 10^−3^	9.20 × 10^−3^
Pancreatic lesion	5.88 × 10^−3^	6.34 × 10^−3^
Formation of solid tumor	5.90 × 10^−3^	1.74 × 10^−3^

(**A**) Top 10 functions identified from gene promoters methylated in placenta but not in cord blood. (**B**) Functions identified from gene promoters methylated in cord blood but not in placenta. (Only 9 common annotations were found.)

## References

[1] Rondinone O., Murgia A., Costanza J., Tabano S., Camanni M., Corsaro L., Fontana L., Colapietro P., Calzari L., Motta S. (2021). Extensive Placental Methylation Profiling in Normal Pregnancies. Int. J. Mol. Sci..

