# Supplementary figures and images for: Extensive Placental Methylation Profiling in Normal Pregnancies
Source: Int J Mol Sci. 2021 Feb 21;22(4):2136. doi: 10.3390/ijms22042136 (PMC7924820; doi:10.3390/ijms22042136)

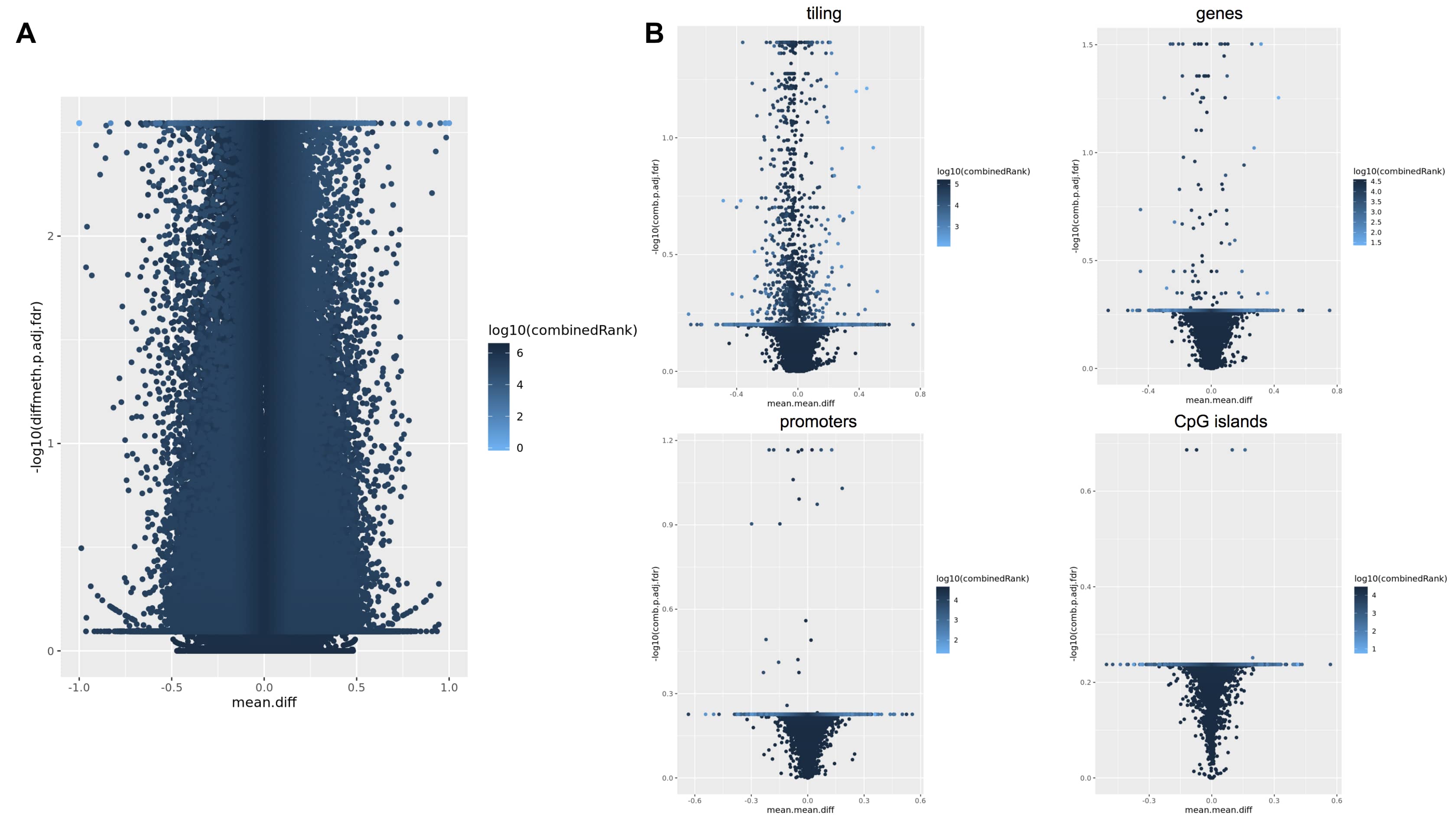

Supplement: Supplementary file 1 [file ijms-22-02136-s001.zip › Supplementary material/supplementary_figure_1.jpg]
